# Believers in pseudoscience present lower evidential criteria

**DOI:** 10.1038/s41598-021-03816-5

**Published:** 2021-12-21

**Authors:** Javier Rodríguez-Ferreiro, Itxaso Barberia

**Affiliations:** 1 Departament de Cognició, Desenvolupament y Psicologia de la Educació, Universitat de Barcelona, Barcelona, Spain; 2 Institut de Neurociències, Universitat de Barcelona, Barcelona, Spain; 3 Grup de Recerca en Cognició i Llenguatge, Universitat de Barcelona, Barcelona, Spain

**Keywords:** Psychology, Human behaviour

## Abstract

Previous studies have proposed that low evidential criteria or proneness to jump to conclusions influences the formation of paranormal beliefs. We investigated whether the low evidential criteria hypothesis for paranormal beliefs extends to a conceptually distinct type of unwarranted beliefs: those related to pseudoscience. We presented individuals varying in their endorsement of pseudoscientific beliefs with two hypothesis testing tasks. In the beads task, the participants were asked to decide from which of two jars containing different proportions of colored beads they were collecting samples. In the mouse trap task, they were asked to guess which rule determined whether a participant-controlled mouse obtained a piece of cheese or was trapped. In both cases, the volunteers were free to decide when to stop collecting evidence before completing the tasks. Our results indicate that, compared to skeptics, individuals presenting stronger endorsement of pseudoscientific beliefs tend to require less evidence before coming to a conclusion in hypothesis testing situations.

## Introduction

The study of the cognitive basis of paranormal beliefs (those which, if genuine, would be in conflict with basic principles of science^1^) has received certain attention in the last few decades. For instance, Brugger and Graves^2^ argued that believers in the paranormal present lower evidential criteria than non-believers, which makes them more likely to accept paranormal explanations for everyday phenomena. In their view, individuals vary in their disposition to accept or reject given explanatory accounts of real-life events. A person prone to believe in the paranormal may consider that a mere coincidence between events (e.g., thinking about a real-life event and that event occurring) is compelling enough to believe that the two events are causally related. In contrast, a more skeptical individual may need more instances of cooccurrence to end up endorsing the causal belief.

These authors tested the low evidential criteria hypothesis by presenting volunteers varying in their endorsement of paranormal beliefs with a “differential reinforcement of low rates of responding task”^3^ designed to induce superstitious behavior. The participants completed 100 trials of a video game-like task in which they were asked to discover the rule determining whether a mouse, controlled by the participant, got a piece of cheese or not. After finishing the task, the participants were presented with different possible rules (i.e. hypotheses). The volunteers had to indicate which of them they had considered during the task (we will call these rules “considered rules”), and which they thought to be actual rules determining the success retrospectively (we will call these rules “claimed rules”). Compared to skeptics, paranormal believers in Brugger and Graves’ study reported testing fewer hypotheses while completing the task. Furthermore, they also ended up believing in more hypotheses regarding the mouse’s success. These results were considered to support the low evidential criteria hypothesis.

The tendency to require less evidence in hypothesis testing situations is directly related to what in more recent times has come to be called a “jump-to-conclusions” bias which, according to Irwin, Drinkwater and Dagnall^4^ results from “the tendency to draw an inference on the basis of very limited information”. Analysing the participants’ performance in an experimental setting known as the beads task^5^, Irwin et al.^4^ observed that believers in the paranormal presented a stronger jump-to-conclusions bias than skeptics (see also Irwin, Dagnall, & Drinkwater^6^ for similar results with self-reported measures of the jump-to-conclusions bias). In the beads task, volunteers are asked to decide from which of two jars they are drawing beads. They are informed that the two jars have different proportions of beads of two different colors. For instance, one of them contains more blue than red beads and the other one contains more red than blue beads. The participant cannot see the inside of the jar, but is allowed to pick beads one by one until reaching a decision. In their study, Irwin et al.^4^ found that believers in paranormal phenomena tended to draw fewer beads than non-believers before completing the task.

Paranormal beliefs are considered to be one specific type of epistemically unwarranted belief, among which we find the closely related but conceptually distinct category of pseudoscientific beliefs^7^. The definition of pseudoscience is still subject to controversy^8^. Although other relevant elements have been proposed (see the discussion of the doctrinal component by Hansson^9^) for many authors the identification of an activity as pseudoscientific relies on two criteria^10^: (a) it is not science, (b) it is presented to create the impression that it is, indeed, science. Paranormal and pseudoscientific beliefs are known to correlate with each other^7,11^, suggesting that they could be influenced by similar cognitive biases. Nevertheless, whether that is actually the case is still to be determined.

In a recent study, individuals showing a more pronounced jump-to-conclusions bias in the beads task also developed a stronger causal illusion in a contingency learning task^12^. Causal illusion refers to the impression that two events (e.g. drug intake and healing from a disease) are causally related when they are actually non-contingent^13^. The observation of an association between the jump-to-conclusions bias and causal illusion is interesting for our study because endorsement of pseudoscientific beliefs has also been associated with a greater tendency to develop causal illusions^14^. In fact, causal illusions can be considered a laboratory model of the emergence of pseudoscientific beliefs^15^.

The aim of this study is to determine whether previous observations indicating lower evidential criteria in believers in the paranormal can also be generalized to individuals endorsing pseudoscientific beliefs. Our hypothesis is that individuals with a reasoning strategy leading them to require limited information before drawing an inference in neutral experimental contexts are also prone to endorse pseudoscience in their everyday lives.

In a first experiment, we studied the possible association between belief in pseudoscience and low evidential criteria by means of the beads task. In an attempt to replicate previous observations by Irwin et al.^4^, we also introduced a paranormal beliefs questionnaire. Following their results, we expected individuals with stronger endorsement of pseudoscientific beliefs to require a smaller number of trials before completing the task.

In a second experiment, we addressed the low evidential criteria hypothesis with regards to belief in pseudoscience by presenting volunteers varying in their endorsement of pseudoscientific beliefs with the mouse trap task. Again, we also included a measure of paranormal beliefs in order to replicate previous results obtained in relation to magical ideation. Importantly, and different from the procedure designed by Brugger and Graves^2^, we allowed the participants to decide how many trials they completed before they finished the task. We believe this approach provides a more direct measure of the amount of evidence required by the participants before they settle on a hypothesis. In consonance with the results observed by Brugger and Graves^2^, we expected participants endorsing pseudoscientific (and paranormal) beliefs to consider fewer hypotheses during the task and end up believing in more hypotheses at the end of the task than volunteers with lower scores on unwarranted beliefs questionnaires. Moreover, we expected participants with higher scores on the unwarranted beliefs questionnaires to require less evidence during the task before reaching to a conclusion, as reflected by a reduced amount of completed trials.

## Experiment 1

### Methods

#### Participants

A group of 59 volunteers took part in the experiment (55 females, mean age = 20.48, SD = 3.01) in exchange of course credits. They were all Psychology students naïve to the task. All data were gathered anonymously. We obtained written online informed consent from all the volunteers before their participation in the study. The University of Barcelona's Bioethics Commission approved the study protocols (Institutional Review Board 00003099). All methods were carried out in accordance with relevant guidelines and regulations.

### Materials and procedure

We constructed an online beads task through Qualtrics (www.qualtrics.com) with the materials provided by Moreno-Fernández et al.^12^. The procedure was based on that of Moreno-Fernández et al.^12^ and Ross et al.^16^. At the beginning of the experiment, participants were asked not to jot down notes during the task. Then, the task was explained. They were presented with two jars, one of them containing 60 blue beads and 40 red beads (mainly blue jar), the other containing 60 red beads and 40 blue beads (mainly red jar). The volunteers had to guess which of the two jars had been poured into a box from which they could take beads one by one. After each draw, they were given the opportunity to provide an answer and end the task, or to continue taking beads. We presented all the volunteers with the same fixed 50 beads color sequence used by Moreno-Fernández et al.^12^ and Ross et al.^16^: (b = blue, r = red) b, r, r, b, b, r, b, b, b, r, b, b, b, b, r, r, b, r, r, b, b, r, b, b, r, b, b, b, r, r, b, r, r, b, b, b, b, r, b, b, r, r, r, r, b, b, r, b, b, b, which extends the 20-beads sequence used previously by Garety et al. ^17^. The dependent variable was the number of beads taken before giving an answer.

After completing the beads task, the participants responded to the Pseudoscience Endorsement Scale (PES)^14^ and the Sheep-Goat Scale (SGS)^18^, in that order. The PES consists of 20 items aimed to assess the endorsement of pseudoscientific beliefs (e.g., “Listening to classical music, such as Mozart, makes children more intelligent.”; “The manipulation of energies bringing hands close to the patient can cure physical and psychological maladies.”. Participants responded by means of a Likert-like scale ranging from 1 (i.e., “Strongly disagree”) to 7 (i.e., “Strongly agree”).

The SGS comprises 18 items aimed to assess endorsement of paranormal beliefs (e.g., “I have had at least one dream that came true and which (I believe) was not just a coincidence”; “I believe that it is possible to gain information about the future before it happens, in ways that do not depend on rational prediction or normal sensory channels”). For each item, the volunteers selected one of three options: false (0 points), uncertain (1 point) or true (2 points). The final score ranges from 0 to 36. We translated the original English version of the SGS into Spanish following common translation and back-translation procedures. First, a Spanish speaker of advanced English proficiency translated the English version into Spanish. Then, an English-native bilingual translator back-translated the Spanish version. The two translators discussed the minor differences revealed by comparing the two versions until they reached an agreement.

At the end of the experiment participants stated their sex and age.

### Results

The full dataset is stored at Open Science Framework repository (OSF, https://osf.io/xvmkn/). We analysed our results with JASP^19^. Participants drew a mean of 12.03 beads, SD = 8.71, before completing the task. Reliability analyses of the responses of the unwarranted beliefs questionnaires indicated adequate reliability values for both the PES, mean = 3.01, SD = 1.05, ω = 0.92, and the SG scale, mean = 0.5, SD = 0.41, ω = 0.88.

**Figure 1:**
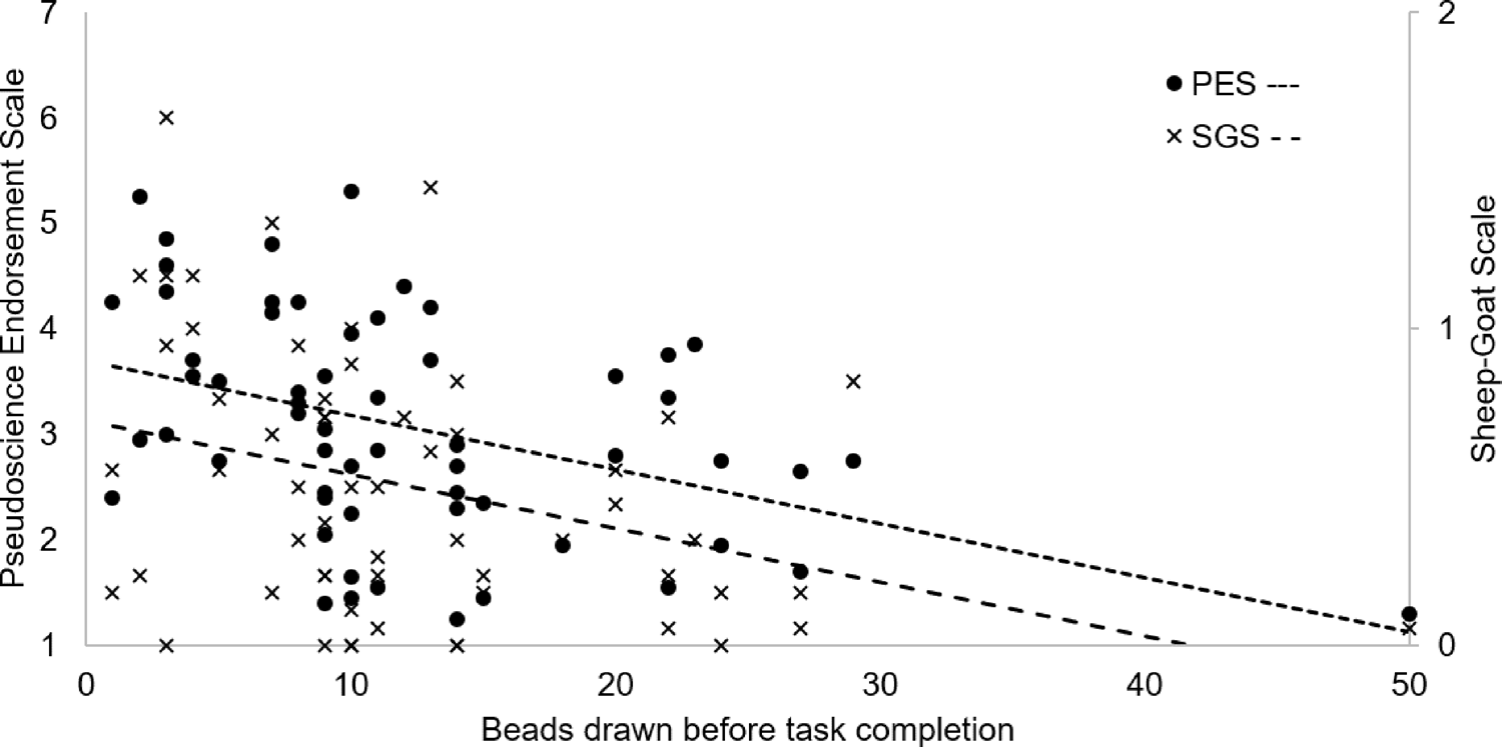
Scatter plot displaying scores on the Pseudoscientific Beliefs scale and Sheep-Goat scale in relation to the number of beads drawn before task completion.

Pearson correlation analyses indicated significant negative associations between the number of beads drawn during the beads task and scores obtained on the pseudoscientific belief questionnaire, *r* =  − 0.421, *p* < 0.001. Besides frequentist analyses, we conducted Bayes factor analyses, which provide an intuitive interpretation of the credibility of the observed effects. A Bayesian correlation analysis indicated that the present data were 36 times more likely given the alternative hypothesis (i.e., that both variables correlated) than the null hypothesis (*BF*_*10*_ = 35.633, see Fig. 1). An analogous, though less robust, result was obtained with regards to paranormal beliefs, *r* =  − 0.356, *p* = 0.006, *BF*_*10*_ = 6.756. In order to check whether the extreme response pattern of the only participant who drew the 50 beads was affecting the results, we ran a parallel set of correlations excluding her data. The pattern of results remained unchanged (pseudoscience: *r* =  − 0.368, *p* = 0.004, *BF*_*10*_ = 8.43; paranormal: *r* =  − 0.338, *p* = 0.009, *BF*_*10*_ = 4.386). The data, thus, confirmed the hypothesis that pseudoscientific and paranormal beliefs are associated with a jump-to-conclusions bias.

## Experiment 2

### Methods

#### Participants

A group of 62 Psychology students naïve to the task and different from those who took part in experiment 1 completed the beads task in exchange for course credits (58 females, mean age = 19.58, SD = 2.67). We obtained online written informed consent from all the volunteers before their participation in the study and the data were registered anonymously. The University of Barcelona's Bioethics Commission approved the study protocols (Institutional Review Board 00003099). All methods were carried out in accordance with relevant guidelines and regulations.

### Materials and procedure

The experiment was completed on-line. We programmed an adaptation of the task designed by Brugger and Graves^2^ with Construct2 (www.construct.net). A demo of the task can be downloaded in the OSF repository. A 3 × 3 grid appeared onscreen with the drawing of a mouse in the lower left box and the drawing of a piece of cheese in a trap in the upper right box. The participant was instructed to freely move the mouse using the arrow keys in the keyboard. If the mouse reached the piece of cheese in four seconds or less, the message “The mouse was trapped!” appeared on the screen accompanied by a buzzing sound. If the mouse reached the cheese after four seconds, the message “The mouse got the cheese!” appeared on the screen accompanied by a high-pitched fanfare.

**Figure 2:**
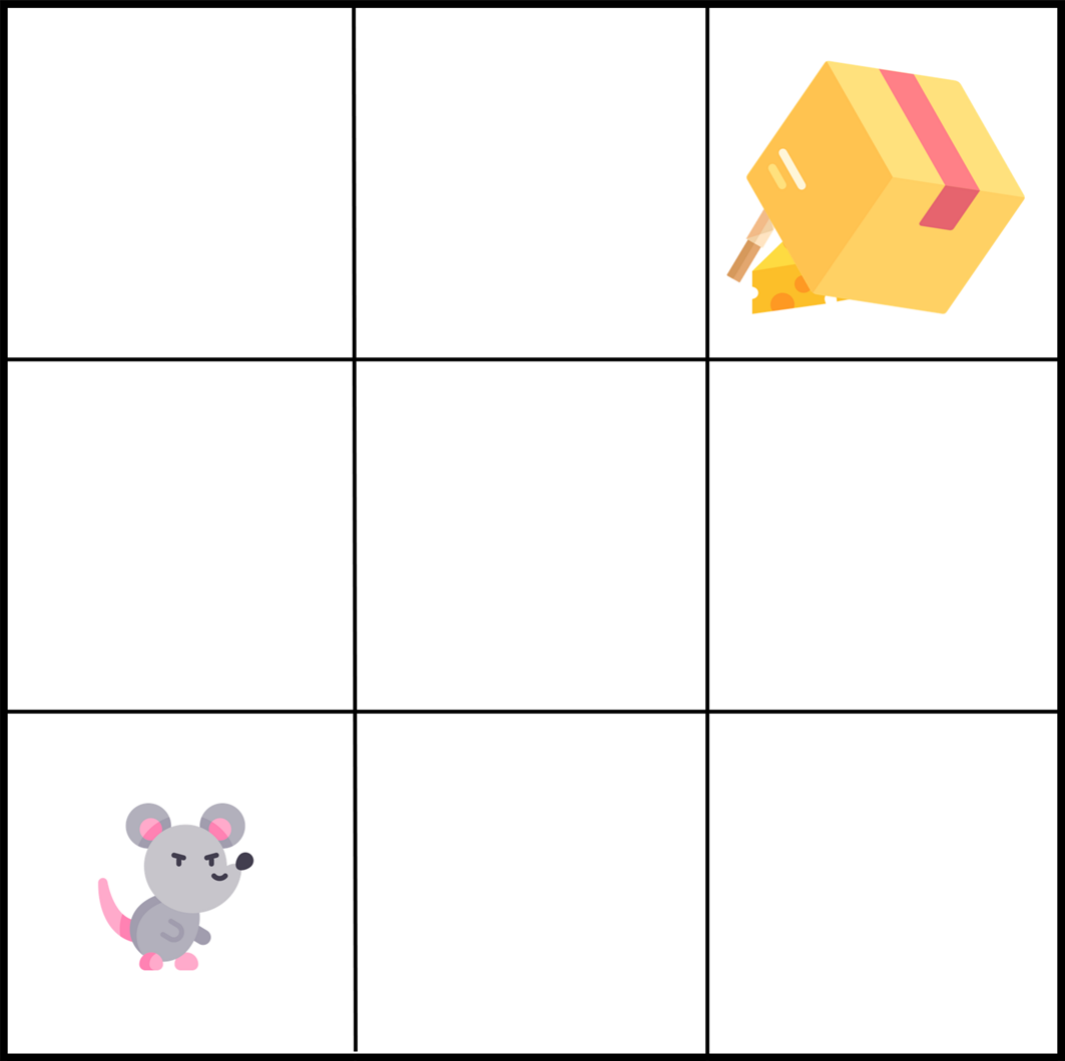
Caption of the initial state of the mouse trap task used in experiment 2. Icons made by Freepik from www.flaticon.com.

Prior to the beginning of the task, the volunteers were instructed to discover the rule that determined whether the mouse obtained the cheese or not. They were informed that they had up to 100 opportunities to complete the task, but, differing from Brugger and Graves’ task, they could stop trying as soon as they thought they had discovered the rule. After every trial, a counter stated how many trials had already been completed and the participants were given the opportunity to either try again (with a maximum of 100 trials) or finish the task. We recorded the total amount of trials completed by each participant. Figure 2 shows a caption of the initial state of the task.

After the mouse trap task had been completed, the participants responded to a series of questions presented through a Qualtrics survey. First, they were asked to write out the rule they thought determined whether the mouse got the cheese or was trapped. Second, they were presented with a list of ten possible rules determining the success of the mouse in the previous task (e.g., “In order to get the cheese, I had to wait a certain amount of time before landing on the trap”; “In order to get the cheese, I had to make sure I visited a particular square”^20^). For each of them, they were asked to state whether they had thought that it could have been relevant at any moment throughout the task (i.e. considered rules). Third, they were presented with the same ten rules again and, for each of them, they were asked to state whether they thought they were actually relevant for the task or not (i.e. claimed rules).

Finally, the participants answered the PES and the SGS, as well as questions regarding their sex and age.

### Results

The full dataset is available at OSF. Seven out of 62 participants discovered the correct rule. On average, the volunteers completed 31.82 trials, SD = 34.37, before finishing the mouse trap task. On average, they selected 4.45 rules, SD = 1.71, in the considered rules questionnaire, and 2.8 rules, SD = 1.97, in the claimed rules questionnaire. Reliability values for the pseudoscientific and paranormal beliefs scales were very similar to those obtained in Experiment 1 (PES: mean = 3.3, SD = 1.04, ω = 0.92; SGS: mean = 0.55, SD = 0.41, ω = 0.88).

We conducted Pearson correlation analyses including the amount of trials completed, considered rules and claimed rules, as well as the scores in the pseudoscientific and paranormal beliefs scales. A summary of the results is presented in Table 1. The number of considered rules positively correlated with the number of completed trials, but not with scores in the pseudoscientific or paranormal beliefs scales. The number of claimed rules positively correlated with PES scores (the correlation with SGS scores only approached significance). The number of completed trials negatively correlated with scores in the pseudoscientific and paranormal beliefs scales. PES and SGS scores were positively correlated.

**Table 1:** Summary of correlation analyses between responses in relation to the mouse trap task and scores in the pseudoscientific and paranormal beliefs scales.

	Considered rules *r* (BF_10_)	Claimed rules *r* (BF_10_)	Completed trials *r* (BF_10_)	PES *r* (BF_10_)
Claimed rules	0.157 (0.328)			
Completed trials	0.317* (3.411)	− 0.233 (0.808)		
PES	− 0.201 (0.524)	0.343** (6.034)	− 0.394** (21.456)	
SGS	− 0.087 (0.198)	0.238 (0.864)	− 0.318* (3.529)	0.581*** (24,886)

*p < .05, **p < .01, ***p < .001.

*PES* Pseudoscience Endorsement Scale, *SGS* Sheep-Goat Scale (paranormal beliefs).

## Discussion

We presented participants varying in their endorsement of pseudoscientific and paranormal beliefs with two tasks aimed to test the low evidential criteria hypothesis. Our prediction was that believers would require less evidence before settling on a hypothesis during both tasks. Our results confirmed the hypothesis, as indicated by significant negative correlations between the amount of trials completed during the mouse trap and beads tasks and scores in the unwarranted belief questionnaires. According to Bayesian analyses, the evidence was especially reliable with regards to the association between the amount of trials completed and endorsement of pseudoscientific beliefs.

Regarding the mouse trap task, and following the original study by Brugger and Graves^2^, we also presented our participants with questionnaires addressing examples of rules they could have assessed during the task (considered rules) or they thought to be actually relevant rules after finishing it (claimed rules). Paranormal believers in Brugger and Graves’ study selected fewer rules in the considered rules questionnaire and more rules in the claimed rules questionnaire than non-believers. Our results, hence, partially replicated those of Brugger and Graves^2^.

On the one hand, and in contrast with the results obtained by Brugger and Graves^2^, our data did not show an association between the amount of considered rules and endorsement of unwarranted beliefs. In our view, differences between the two studies could be due to the type of measure used. In both studies, participants were asked to select the considered rules among a closed set of alternatives. It could be the case that the set of rules offered to the participants in our study did not include all the rules they had considered and, thus, our measure did not adequately reflect their reasoning process.

An open question asking the volunteers to write all the hypotheses they had considered during the mouse trap task might have been more adequate to fully grasp their strategy. Nevertheless, using this kind of question is not without problems, as the responses can be affected by the participant’s willingness to dedicate time to the experiment and write one or more rules (which could be directly related to the time spent completing the mouse trap task itself). Moreover, it introduces noise due to the necessity of response recoding by the experimenters. In any case, according to our data, the amount of trials completed appears to be a robust indicator of the hypothesis testing strategy applied during the task or, at least, of its association with endorsement of unwarranted beliefs.

On the other hand, we replicated the observed significant positive association between scores on the pseudoscientific beliefs questionnaire and the amount of claimed rules. Again, the results were less robust in the case of paranormal beliefs, for which the association with the scores corresponding to the claimed rules questionnaire only approached significance. This result indicates that, despite gathering less evidence during the task, believers ended up endorsing more (erroneous) hypotheses than skeptics.

Our study assumes that humans rely, at least to some extent, on rational assessment of empirical evidence to adopt new beliefs. Nevertheless, this need not be the case. Despite complete lack of empirical evidence to support them, unwarranted beliefs might gain popular support by means of intuitive appeal (i.e., providing “comfortable intuitive representations of the world” ^21^) or even capitalize the cognitive optimum^22^ achieved through the combination of intuitive content and salient, attention-grabbing, minimal counterintuitive inconsistencies ^23^. Furthermore, reasons other than strong empirical evidence might also play a role in the spread of unwarranted beliefs. For instance, one can adhere to a belief because it is coherent with prior knowledge or because it comes from a trusted source ^24^.

Moreover, endorsement of different kinds of beliefs might depend on different cognitive strategies. For example, Metz et al.^25^ observed that, when asked to justify their beliefs, evolutionists emphasize empirical evidence and scientific consensus whereas creationists emphasize intuition or “knowledge from the heart” and (religious) authority. In the same vein, and directly related with our study, McPhetres et al.^26^ showed that, when religious and non-religious participants were asked to rate how many times they expected an effect to be repeated for them to consider the proposed explanatory claim to be true, religious participants requested fewer repetitions when the effect was attributed to prayer compared to when it was attributed to scientific methods.

Our study has practical implications for the design and implementation of interventions aimed to reduce the presence of unwarranted beliefs in the population. If endorsement of these kinds of belief are rooted in suboptimal evidential standards, then we could expect that training individuals in adequate sampling strategies would induce re-assessment of previously acquired beliefs and influence the acquisition of new ones. In our view, an intervention consisting of training-in-bias^27^, based on tasks like those used in our study, and training-in-rules^27^, focusing on teaching basic principles of sampling strategies and sample variability, both abstractly and with concrete examples^28^, could have an impact over the presence of paranormal and pseudoscientific beliefs.

An important limitation of our study is related to the nature of our sample. It should be taken into account that our participants were young university students which might not fully represent the general population with regards to endorsement of unwarranted beliefs. In this sense, further studies should test whether our results would be replicated with a more heterogeneous and representative sample. Additionally, the fact that they were specifically Psychology students, could also be of concern. Although the volunteers were all in the first or second year of the Psychology degree (students in their third year, in which topics central to the study are directly discussed, or higher were prevented from participating) some of them could have already acquired some knowledge of experimental design and hypothesis testing procedures, which could have affected their performance in the two tasks. With this in mind, we conducted complementary sets of analyses conditioning the correlation between the different dependent variables on participants’ age. The pattern of results remained virtually unaffected by the inclusion of this covariate, suggesting that, although we cannot completely rule out this possibility, knowledge acquired during the degree did not affect the results.

Another limitation of our research, which directly stems from the aforementioned, is related to imbalance between men and women in the sample. In Brugger and Graves’^2^ study, in which half of the participants were men and half women, no differences were observed according to this variable. In the study by Irwin et al.^4^ the sample was also composed mainly of women but the authors did not take this variable into account. In our study, parallel sets of analyses including only the data from women yielded a pattern of results similar to that obtained with the whole sample in both experiments. However, the fact that our sample was mostly composed of women means that our study cannot rule out possible gender-related differences with regards to the association between hypothesis testing strategies and endorsement of unwarranted beliefs.

In conclusion, our study provides evidence indicating that endorsement of unwarranted beliefs is associated with low evidential criteria, leading individuals to test fewer hypotheses before settling to one of them, hence showing a jump-to-conclusions bias. We replicated the general pattern of results observed by Brugger and Graves^2^ and by Irwin et al.^4^ in relation to paranormal beliefs, widening their implications to the field of pseudoscientific beliefs, which are more relevant in contemporary society. Besides, our study also presents an important methodological contribution regarding the mouse trap task, as we show that allowing volunteers to stop the hypothesis testing task at will and measuring the amount of trials completed might provide an objective and robust measure of their reasoning process.

## Data Availability

The datasets generated and/or analysed the current studies are available in the Open Science Framework repository (https://osf.io/xvmkn/).
